# Chlamydia Trachomatis Tonsillopharyngitis

**DOI:** 10.1155/2012/736107

**Published:** 2012-09-20

**Authors:** Özmen Öztürk, Hüseyin Seven

**Affiliations:** Department of Otorhinolaryngology and Head and Neck Surgery, School of Medicine, Istanbul Medipol University, 34718 Istanbul, Turkey

## Abstract

Reports about the extragenital spread of *Chlamydia trachomatis (CT)* to oropharynx are limited. We report a male patient with progressive tonsillopharyngitis resistant to amoxicillin/clavulanic acid therapy. The patient presented 9 days after an orogenital and oroanal sexual intercourse with a female sex worker. The microimmunofluorescence revealed *CT* tonsillopharyngitis, and after completing a one-week course of *doxycycline*, the patient recovered completely. More cases of *CT* tonsillopharyngitis may be revealed if attention is paid to an association of sexual activity with enduring tonsillopharyngitis.

## 1. Introduction

Sexual habits have changed over the preceding years, with an increase in the prevalence of sexually transmitted diseases (STDs). The etiological relationship of the most widespread STD pathogen, *Chlamydia trachomatis* (*CT*), to reproductive tract has long been established, but the distribution to extragenital sites (e.g., respiratory tract, oropharynx, middle ear, and eye) appears to be infrequent, and its correlation with the sexual practice still requires to be investigated [1, 2]. In this paper, a male patient with *CT* tonsillopharyngitis in relation to an orogenital and oroanal sexual intercourse is presented. 

## 2. Case Report

**Figure 1:**
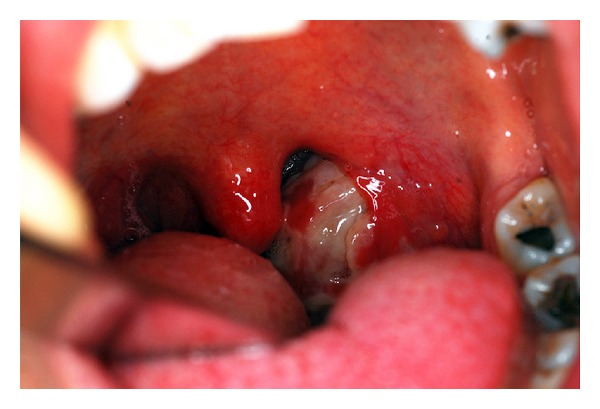
*Chlamydia trachomatis* tonsillopharyngitis. There is generalized pharyngeal and tonsillar hyperemia with diffuse purulent exudate on the left tonsil and swollen anterior pillars and uvula.

A previously well 37-year-old male patient presented with sore throat, odynophagia, high fever, and malaise for a period of one week. The patient reported that he had been using amoxicillin/clavulanic acid 1000-mg/62.5-mg tablet, twice daily, for a period of 5 days, but the clinical symptoms were deteriorating. An otorhinolaryngologic examination revealed hyperemic and congested oropharynx and tonsils with diffuse exudate predominantly on the left tonsil (Figure 1) and bilateral Level I-II cervical lymphadenopathies. The patient was immediately hospitalized, and penicillin G potassium was administered with a dose of 4 million-units q4 hrs. Throat culture for group A *β*-hemolytic streptococci showed normal flora. Monospot test for heterophile antibodies was negative. Chest X-ray and upper abdominal ultrasound examinations were within normal limits. A complete blood count showed leucocytosis (11.29 × 10^3^/uL), with 73.2% neutrophilia and 12.4% lymphopenia. On the 4th day of the hospitalization, the patient gave a history of a sexual intercourse with a female sex worker (FSW), 9 days before hospitalization. The patient admitted that coitus did not take place, the sexual act was predominantly with cunnilingus and anilingus, and mutual masturbation with manual contact. On the 6th day, laboratory work-up for a STD (e.g., gonorrhea, syphilis, and chlamydia) declared a positivity for Chlamydial IgM with microimmunofluorescence. Total urine count showed rare leucocytes, and urine culture was negative. Medical therapy was changed to doxycycline 100 mg tablets, twice daily, on the 6th day of hospitalization. A pronounced symptomatic improvement was seen in the first 48 hours of the new drug regimen, the signs of tonsillopharyngitis settled down, and the patient was discharged on the 8th day. After completing a one-week course of doxycycline, the patient recovered completely.

## 3. Discussion

*CT* has been cultured from the oropharynx in asymptomatic people who have engaged in receptive orogenital sexual intercourse. In homosexual men, an evidence of pharyngeal *CT* infection was found with a prevalence of 1.5% [1]. In heterosexuals, *CT* has been isolated from the pharynx in 3.7% of men and 3.2% of women [2]. Although opposed by many other studies, Komaroff et al. have demonstrated that *CT* may be a cause of pharyngitis in adults, accounting for approximately 20% of the cases of his study [3]. In a study on chimpanzees, a pharyngeal infection was produced only in the situation that a high amount of *CT* inclusion-forming units to the pharynx mucosa was inoculated [4]. This finding showed that a strong *CT* inoculation to the pharyngeal mucosa might induce an infection. Asymptomatic FSWs may act as a reservoir of *CT* infection, and in our paper, the patient's act of orogenital and uororectal sexual intercourse with a FSW provided the required load of *CT* to oropharynx. The presence of *CT* in tonsillar crypts was also investigated in order to determine the relationship of *CT* to a tonsillar infection, and in approximately a quarter of the cases, *CT* was recovered from tonsillar crypts [5]. 

It is almost impossible to identify *CT* tonsillopharyngitis by clinical symptoms and signs alone. Sexual exposure to an infected partner is accepted as an indication for obtaining specific investigations. Throat culture, the most definitive method of diagnosis, is performed by staining the pharyngeal specimen with *CT* fluorescein-labeled monoclonal antibody. Microimmunofluorescence from blood serum allows identification of specific antibodies for the serodiagnosis. Tetracycline and erythromycin have formed the basis of treatment, and sex partners should also be notified to prevent the spread of infection. 

 The association of idiopathic tonsillopharyngitis with *CT,* particularly after orogenital sexual intercourse, may be more common than recognized. More cases may be revealed if attention is paid routinely to an association of sexual activity with persistent tonsillopharyngitis.
